# Effect of Clinically Used Microtubule Targeting Drugs on Viral Infection and Transport Function

**DOI:** 10.3390/ijms23073448

**Published:** 2022-03-22

**Authors:** María Ángela Oliva, Carlota Tosat-Bitrián, Lucía Barrado-Gil, Francesca Bonato, Inmaculada Galindo, Urtzi Garaigorta, Beatriz Álvarez-Bernad, Rebeca París-Ogáyar, Daniel Lucena-Agell, Juan Francisco Giménez-Abián, Isabel García-Dorival, Jesús Urquiza, Pablo Gastaminza, José Fernando Díaz, Valle Palomo, Covadonga Alonso

**Affiliations:** 1 Unidad BICS, Centro de Investigaciones Biológicas Margarita Salas, Consejo Superior de Investigaciones Científicas, Ramiro de Maeztu 9, 28040 Madrid, Spain; marian@cib.csic.es (M.Á.O.); carlota.tosat@cib.csic.es (C.T.-B.); barrado.lucia@inia.es (L.B.-G.); francesca.bonato@cib.csic.es (F.B.); beatriz.alvarez@cib.csic.es (B.Á.-B.); rebeca.paris@cib.csic.es (R.P.-O.); lucena@cib.csic.es (D.L.-A.); gimenezjf@cib.csic.es (J.F.G.-A.); fer@cib.csic.es (J.F.D.); 2 Centro de Investigación Biomédica en Red de Enfermedades Neurodegenerativas (CIBERNED), Instituto de Salud Carlos III, 28031 Madrid, Spain; 3 Departamento de Biotecnología, Instituto Nacional de Investigación y Tecnología Agraria y Alimentaria (INIA-CSIC), Consejo Superior de Investigaciones Científicas, Carretera de la Coruña km 7.5, 28040 Madrid, Spain; galindo@inia.es (I.G.); isabel.garcia@inia.es (I.G.-D.); jesus.urquiza@inia.es (J.U.); 4 Centro Nacional de Biotecnología, Consejo Superior de Investigaciones Científicas, Calle Darwin 3, 28049 Madrid, Spain; ugaraigorta@cnb.csic.es (U.G.); pgastaminza@cnb.csic.es (P.G.); 5 IMDEA Nanociencia, Faraday 9, 28049 Madrid, Spain

**Keywords:** antivirals, microtubule targeting drugs, SARS-CoV-2, mebendazole-tubulin complex crystal structure

## Abstract

Microtubule targeting agents (MTAs) have been exploited mainly as anti-cancer drugs because of their impact on cellular division and angiogenesis. Additionally, microtubules (MTs) are key structures for intracellular transport, which is frequently hijacked during viral infection. We have analyzed the antiviral activity of clinically used MTAs in the infection of DNA and RNA viruses, including SARS-CoV-2, to find that MT destabilizer agents show a higher impact than stabilizers in the viral infections tested, and FDA-approved anti-helminthic benzimidazoles were among the most active compounds. In order to understand the reasons for the observed antiviral activity, we studied the impact of these compounds in motor proteins-mediated intracellular transport. To do so, we used labeled peptide tools, finding that clinically available MTAs impaired the movement linked to MT motors in living cells. However, their effect on viral infection lacked a clear correlation to their effect in motor-mediated transport, denoting the complex use of the cytoskeleton by viruses. Finally, we further delved into the molecular mechanism of action of Mebendazole by combining biochemical and structural studies to obtain crystallographic high-resolution information of the Mebendazole-tubulin complex, which provided insights into the mechanisms of differential toxicity between helminths and mammalians.

## 1. Introduction

Microtubules (MTs) are involved in cell division through the formation of the mitotic spindle and angiogenesis, and hence, these filaments are the main targets of anticancer treatments. There is an important arsenal of MTs targeting agents (MTAs) of clinical use that have been extensively characterized in terms of pharmacological properties and toxicity [1]. In addition, some MTAs also have applications in inflammatory diseases [2] or as anti-helminthics [3].

**Figure 1:**
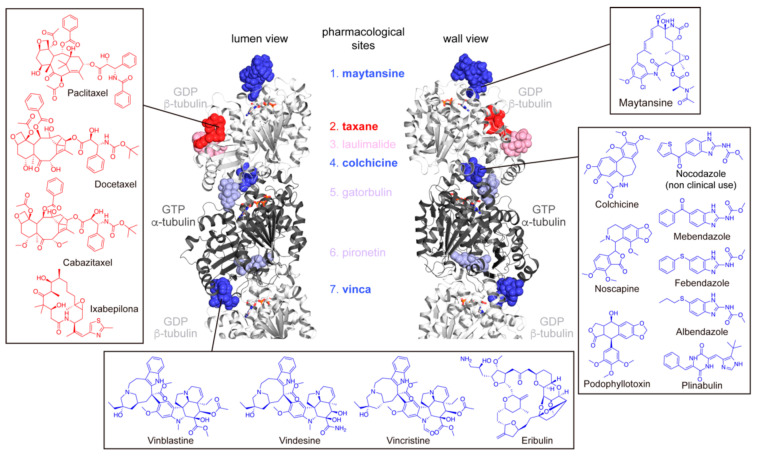
Tubulin pharmacological binding sites. MT lumen (left) and wall (right) views of α,β-tubulin heterodimer (α-tubulin GTP-bound in grey, β-tubulin GDP-bound in white) in ribbon representation. The seven known pharmacological binding sites are color-coded: blue for MDAs, red for MSAs, dark colors for sites targeted by clinically used drugs and, light colors for sites targeted by compounds not of clinical use. In the same color code are the structure and name of the MTAs assayed in this study, which are grouped by targeted binding site.

MTs are polymers built from αβ-tubulin heterodimer, which has seven known pharmacologically active sites (Figure 1) [4,5]. The interaction of compounds with these sites mediate MTs’ stabilization (MT stabilizing agents, MSAs) or prevent MTs’ assembly (MT destabilizing agents, MDAs). MSAs function as staples preventing GDP-tubulin disassembly either by reinforcing the lateral or the longitudinal interactions within the MT lattice (taxane and laulimalide sites) [6,7]. Instead, MDAs prevent the curved-to-straight structural change necessary for the assembly into MTs (colchicine, vinca, and gatorbulin sites [5,8,9]). Alternatively, two more sites for MDAs are involved in blocking tubulin axial interfaces essential in the longitudinal MT growth (maytansine domain [10] and pironetin site [11,12]). Current clinically approved MTAs bind to four of the seven known tubulin binding sites (Figure 1, dark colors) and might function as MSAs, which are Taxol^®^ (paclitaxel; PTX), Taxotere^®^ (docetaxel; DTX), Jetvana^®^ (cabazitaxel; CTX), and Ixempra^®^ (ixabepilone; EPO), or MDAs, like colchicine (COL), noscapine (NOS), podophylotoxin (PPT), mebendazole (MBZ), albendazole (ABZ), febendazole (FBZ), plinabulin (PLIN), Velban^®^ (vinblastine; VBL), Oncovin^®^ (vincristine; VCR), Endesine^®^ (vindesine; VDS), Halaven^®^ (eribulin; ERIB), and Kazyla^®^ (maytansine; MAYT) (list of FDA-approved works https://www.fda.gov/Drugs, last accessed on 19 March 2022). Most of these drugs are in the WHO Essential Medicines List (World Health Organization Model List of Essential Medicines, 21st List, 2019. Geneva: World Health Organization; 2019), and hence, they are therapeutic alternatives that are affordable and available worldwide.

Importantly, MTs are also an essential part of multiple biological processes, being part of the cellular scaffold and representing the roads along which the cellular long-distance traffic occurs. Indeed, the MT-associated motor proteins, kinesins, and dyneins, recognize MTs’ polarity and transport cargos along them in both directions [13,14]. Remarkably, viruses often take advantage of the host transport machinery that functions through the cytoskeleton for their active movement into the cell. Indeed, viruses frequently depend on an intact MT network and interact with the cytoskeleton at multiple stages of their replication cycles. Some viruses associate directly with the MT-dependent motors for the transport of intact virions, capsids, or any other viral components (individual viral proteins, RNA, etc.), towards the replication sites, and for the exit from replication sites to the plasma membrane. This motion should be similar to any other movements linked to MT motors [15]. However, several viruses are transported along MTs inside endosomes with the characteristic vesicular movement that targets either kinesins or dyneins upon endosomal maturation [16]. Indeed, it has been suggested that viral capsid proteins could direct the regulation of motor proteins [17]. Finally, non-motile MT-associated proteins (MAPs), including MT plus-end tracking proteins (+TIPs), can also conduct viral trafficking in infected cells [18].

We hypothesized that pharmacological modulation of MTs could potentially compromise virus replication and spreading, and evidence their potential as broad-spectrum antivirals. On the other hand, as the intracellular transport machinery is essential in cells, any pharmacological intervention must be tightly controlled to counteract viral infection without compromising key physiological cellular functions. Importantly, the huge gap in timescale between the diseases often targeted by these MTAs and viral infections (months or even years in cancer vs. hours or days in acute viral infections) indicates that it should be possible to find a therapeutic window based on short-term or lower dose administration without reaching undesired secondary effects [19]. Moreover, MTAs are effective when used in combination with other chemotherapeutic drugs [20], suggesting that they could be exploited as broad-range therapeutics that enhance the effect of other more specific antivirals. Therefore, we explored the ability of clinically approved MTAs to inhibit viral replication on five different viruses, representing both the RNA and DNA virus families, and the compounds capacity to impair intracellular transport. In addition, we investigated the underlying molecular mechanism of one of the most efficient drugs (MBZ) applying high-resolution structural studies, which contributes to direct site-specific future drug development in this area through the most efficient tubulin binding site. Altogether, our results support MTs as promising cellular targets in viral diseases and contribute to the future potential of MTAs as wide-spectrum antivirals.

## 2. Results

### 2.1. Effect of MTAs in Viral Infection

We selected 16 commercially available MTAs, including 15 in clinical use, so they could be repositioned in case of effective activity, and five different viruses, comprising ssRNA and dsDNA and affecting humans and animals. Among ssRNAs, we studied human common cold coronavirus (HCoV), the pandemic SARS-CoV-2 coronavirus, both enveloped viruses with positive-sense single stranded RNA genome. We also tested vesicular stomatitis virus (VSV), which is a negative-strand RNA virus belonging to the Rhabdoviridae family. Among dsDNA viruses, we analyzed the poxvirus vaccinia (VACV) and African swine fever virus (ASFV). We expected that the antiviral effect of the compounds would be variable, depending on the times of infection analyzed and the relative dependence on MTs of each virus according to their life cycles.

**Figure 2:**
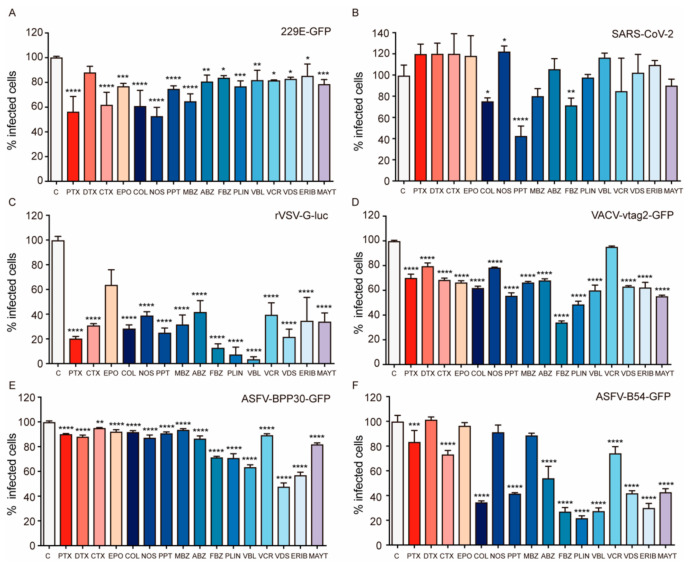
Effect of MTAs in viral infectivity. Cells were pretreated with either compounds or the vehicle DMSO for one hour before infection. The percentages of infection have been normalized to DMSO values. (**A**) Huh-7 cells infected with 229E-GFP detected by flow cytometry after 24 h of infection. (**B**) Antiviral effect on SARS-CoV-2 of compounds at optimal doses, PTX (25 nM), DTX (12.5 nM), CTX (0.5 µM), EPO (12.5 nM), COL (100 nM), NOS (50 µM), PPT (100 nM), MBZ (2 µM), FBZ (2 µM), ABZ (2 µM), PLIN (2 nM), VBL (100 µM), VDS (6 nM), VCR (400 nM), ERIB (2.5 nM), and MAYT (20 nM). (**C**) Infection percentages of VSV-G-luc in Vero-E6 cells detected after 24 h of infection by Relative Light Units (RLU) in a luminometer. (**D**) Infected Vero cells with vtag2GFP detected by flow cytometry after 24 h of infection. (**E**) Infected Vero cells with BPP30GFP detected by flow cytometry after 24 h of infection. (**F**) Infected Vero cells with B54GFP-2 detected by flow cytometry after 24 h of infection. Data presented as mean. Error bars indicate S.D. from three (**A**,**C**–**F**) or two (**B**) independent experiments (one-way ANOVA with Bonferroni post-test). Statistically significant differences are indicated by asterisks (**** *p* < 0.0001, *** *p* < 0.001, ** *p* < 0.01, * *p* < 0.05), n = 9 (**A**,**C**–**F**), n = 6 (**B**).

We first characterized the effect of the compounds studied on human coronavirus infection in Huh-7 cells at concentrations that allowed over 80% cell viability in this particular cell line (see Materials and Methods). For detection purposes, we used a virus whose ORF4 was replaced by EGFP (229E-GFP) (Figure 2A). We found that overall MSAs and MDAs reduced 229E-GFP infectivity significantly after 24 h post-infection (hpi), confirming our initial hypothesis and suggesting that MT transport is a key target for antiviral therapy. Among the compounds studied, those targeting the colchicine domain were more active, and some of them induced viral infection inhibition at ranges between 35 and 50%. Subsequently, we analyzed the effect of these drugs on the infection of the pandemic β-coronavirus SARS-CoV-2, which was included in a large screen of potentially active drugs. In this case, we used human carcinoma epithelial lung cells A549 expressing the SARS-CoV-2 receptor hACE2. These cells were generated in the laboratory by transduction of A549 cells with a retroviral vector expressing hACE2 as described [21]. Cells were first pretreated for one hour with compounds, then infected with SARS-CoV-2, and finally, incubated for 48 h before analyzing their effect on virus infection. In this screen, we used three different drug concentrations, one of them at the estimated CC50 values determined in the drug toxicity assays, and the other two at 0.5 and 2 times these CC50 values (Figure S1). The most effective tolerable drug concentration for each compound was depicted in Figure 2B and, among the MTAs tested, we found that again the colchicine domain binders were the most successful drugs affecting viral replication. Amongst these compounds, PPT achieved the most active inhibition of SARS-CoV-2 infection (60%), followed by FBZ and COL.

We also investigated the effect of these drugs on the infectivity of other unrelated ssRNA viruses, and we analyzed VSV infection in Vero E6 cells (Figure 2C). In these experiments, the doses of drugs used were higher due to the resistant profile of this cell line. Interestingly, the effect on virus infection was remarkably different. Both MSAs and MDAs showed significant antiviral activities (except the stabilizer EPO). Colchicine domain drugs decreased VSV infection from 50 to 80%, while vinca domain drugs VBL and VDS decrease virus infection by 90 and 80% respectively. Also, MAYT was active in reducing virus infection by 40% (Figure 2C).

We next evaluated the dsDNA VACV infection, which is known for its dependence on MTs during its viral cycle, relying on both dynein and kinesin motors [22,23,24]. For detection purposes, we used a recombinant VACV expressing GFP under an early/late promoter, named vtag2GFP (Figure 2D). Remarkably, both MSAs and MDAs induced only a moderate effect (<30% of reduction of viral infection). Only colchicine domain ligands FBZ and PLIN were able to reduce by 40 and 50% the viral infection.

Finally, we selected another DNA virus in which MT motor proteins are also known to be essential, the ASFV [25,26], and we monitored the effect of MTAs on early and late infection events. To do so, we used the recombinant virus BPP30GFP, which expresses the GFP protein under the control of early p30 promoter (Figure 2E) and the recombinant B54GFP that expresses the GFP protein fused to the late viral protein p54 (Figure 2F).

Of note is that the results of the inhibition of infectivity were dependent on the time points of evaluation of the infection, considering that both early and late times could be affected. Remarkably, the pharmacological modulation of MTs had a higher effect on late ASFV infection events compared with early infection. In this case, vinca domain compounds inhibited up to 70% of infection (B54GFP), while those binding to the colchicine domain also presented a significant impact in viral infection. Instead, we only detected moderate antiviral activities of MSAs compounds, highlighting CTX (Figure 2F). The effect of these drugs in the early infection showed that the most active compounds were also vinca alkaloids such as ERIB, VBL, and VDS (up to 50% reducing virus infection), whereas colchicine domain compounds FBZ and PLIN only inhibited 30% ASFV infectivity. In contrast, the other compounds analyzed showed lower antiviral activity (Figure 2E).

### 2.2. Effect of MTAs in the Movement of Cellular Motors

**Figure 3:**
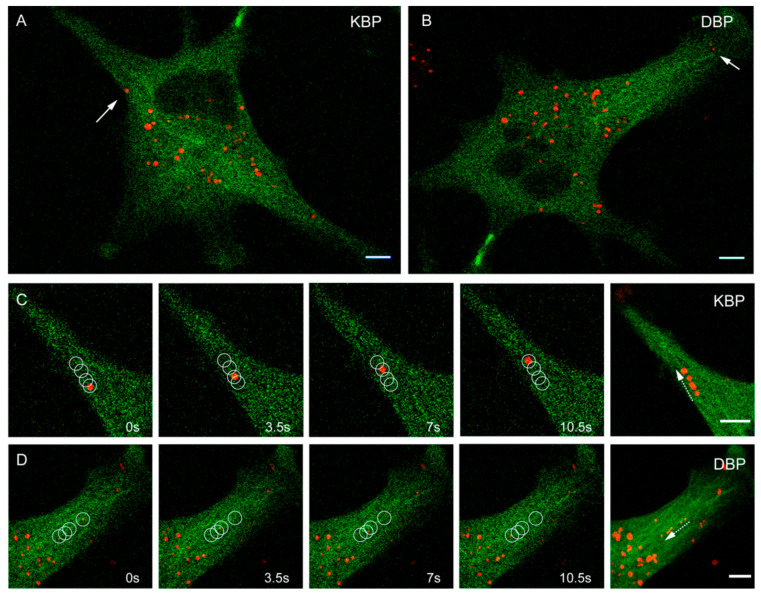
Representative confocal images of AHRTG cells incubated with 2.5 µM KBP (**A**) or DBP (**B**), showing the microtubular network in green and the peptides probes designed as red dots. The white arrows indicate representative particles of each peptide that are further shown in panels ((**C**); KBP) and ((**D**); DBP). These panels show representative time-lapse images at times 0, 3.5, 7, and 10.5 s and superposition of these, of the movement of a KBP and a DBP particle, respectively. The resulting trajectories are indicated as white circles. Scale bar: 10 µm. See also Videos S1 and S2.

The antiviral activity of MSAs and MDAs could result from modifications of the MT intracellular transport function. We generated tools to monitor the movement of molecular motors kinesin and dynein along MTs in cells and analyzed the effect of compounds on this movement. We designed a dynein-binding peptide probe (DBP) based on the binding motive DynPro from the ASFV that had previously been used as an efficient dynein shuttle for gold nanoparticles [27]. For the kinesin binding peptide probe (KBP), we selected a sequence that previously showed specific binding to kinesin 1 light chain [28]. We prepared these peptide probes in a modular manner, including a Cy5 fluorophore at the N-terminal of the peptide and a C-terminal domain of eight arginines (R8) for facilitating entry through the cell membrane. Additionally, we developed the AHRTG cell line, consisting of human A549 lung cancer cells where GFP-α-tubulin is constitutively expressed under a CMV promoter, allowing the direct visualization of the microtubular network in real time. Note that molecular motor interaction with MTs is modulated by the tubulin code [29,30], and therefore, the results obtained should be taken considering that this code is altered in tumoral cells A549 cells (β-tubulin isotype distribution is 80% βI and 20% βIII (own data) vs. mostly βI in non-tumoral lung cells [31]). Incubation of AHRTG cells with either 2.5 µM KBP or 2.5 µM DBP for 15 min allowed in vivo imaging in a confocal microscope recording movies after gentle washing to remove the excess of peptide. We took 4 min movies of frames captured every 1.739 s to analyze the intracellular active transport inside cells (Figure 3, Videos S1 and S2). We classified the movements of particles observed as three types: Brownian motion (BM), constraint motion (CM), and transported motion (TM) (see Materials and Methods).

The behavior of the particles was then described considering the ratio between particles with transported motion and constrained motion (TM/CM), the mean track displacement, and the mean track velocity.

**Figure 4:**
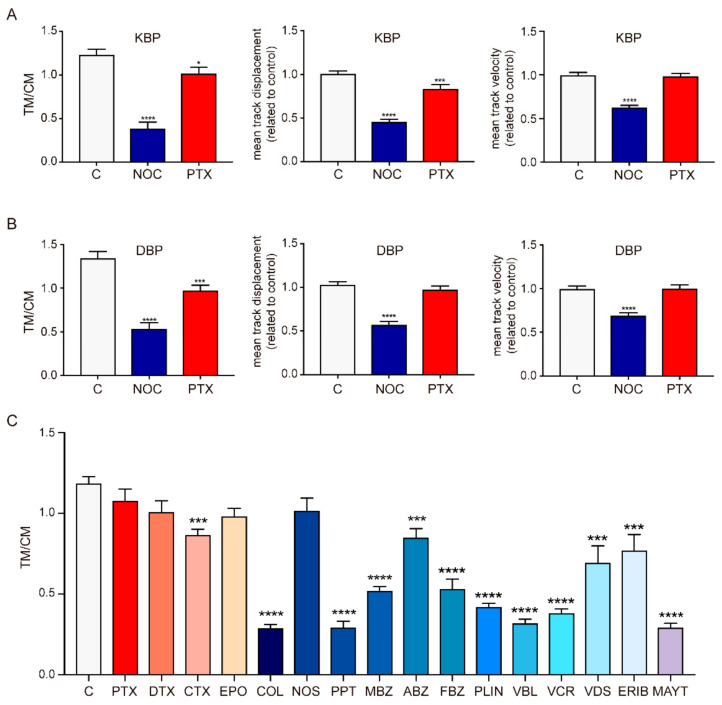
Particle movement parameters measured with KBP (**A**) and DBP (**B**) in the presence of NOC (2.5 µM) and PTX (10 µM) in AHRTG cells. TM/CM (**left**), mean track displacement (**center**), and mean track velocity (**right**) analyzed using the TrackMate plugin in ImageJ and the @msdanalyzer in MATLAB. (**C**) KBP TM/CM measured upon pharmacological treatment with clinically available MTAs. Data presented as mean. Error bars indicate S.E.M. from two independent experiments (one-way ANOVA with Bonferroni post-test). Statistically significant differences are indicated by asterisks (**** *p* < 0.0001, *** *p* < 0.001, * *p* < 0.05), n = 6.

In order to validate these tools for the analysis of the effect of MTAs on the movement of motors, we first compared the movement parameters of the probes in untreated cells with those in the presence of two commonly used MTAs that are well-known to affect MT dynamics: nocodazole (NOC) from the group of MDAs [32] and PTX as an MSA [33]. We initially tested high concentrations of both compounds to determine the effect of a deeply modified MT network on the movement of motor proteins. NOC significantly reduced the network of MTs and we found a diminished mean movement of 68% for KBP and 60% for DBP, shorter mean displacements that decreased by 54% with KBP and 44% with DBP, and lower mean velocities that decreased by 36% with KBP and 30% with DBP (Figure 4A,B). These results indicate that the disruption of the cellular MT network affected the movement monitored by both probes as expected [13,14]. PTX induces a characteristic formation of bundles and, interestingly, this effect had different consequences on the movement of KBP and DBP. The proportion of DBP-transported particles was significantly reduced (27%), while we found no alterations on mean displacement and velocity (Figure 4B). Since taxanes initially bind to a specific region at the MT end [34], inducing MT lattice axial expansion [35], our results support that MT stabilization by PTX might only affect the initial recognition of the MT plus-end by dynein, while once attached to the MT surface, the motion along the filament remains the same. On the other hand, the proportion of KBP-transported particles was slightly reduced (17%) (Figure 4A), while we measured no meaningful changes on the mean velocity, which could be due to the detachment of the motor protein from the microtubular network. Opposite to DBP, we found slight changes on KBP mean displacement that decreased by 16% compared to control experiments, indicating that MT bundling would interfere in the progression of the motor protein, promoting frequent detachments (displacement decreased while velocity was kept constant). Furthermore, the axial expansion induced by PTX in the MT lattice [35] could also affect the processive tandem operation of the two head groups in kinesin, favoring the dissociation of both heads at the same time and consequently, kinesin detachment. We selected KBP for subsequent studies because kinesins presented a more restricted type of movement along MTs in terms of step size and directionality than dynein [36]. Dose-dependent experiments showed that at increasing concentrations of NOC, the effect on KBP motion was higher with lower TM/CM ratio, mean displacement, and velocity (Figure S2A), which denotes that the higher the destabilization of the microtubular network, the smaller the tracking movement of the probe. Note that since MDAs do not bind to assembled MTs but only to unassembled dimers, the influence of the compounds in the transport could be exerted in two ways (which cannot be distinguished at the optical resolution of GFP-labeled MTs in the confocal microscope), by shrinkage of the MTs or by modifications of the structure of the MT ends, which leads to the faulty attachment of the motors to the MT. However, the effect of MT stabilization was less obvious according to the difference between a main stabilization of filaments at low concentrations vs. bundling between filaments at higher concentrations. Interestingly, the proportion of transported particles was only affected at the highest dose (MT’s bundling), a concentration at which the mean track displacement also decreased (Figure S2B). As shown in our initial high PTX dose experiments (Figure 4A), the mean velocity of KBP remained unaltered under the three doses studied.

Next, we explored the effect of all clinically available MTAs on the movement of KBP (Figure 4C and Figure S2C). In order to amplify and detect the short-term effects of the compounds in the motor movement, we used the maximum concentrations allowing cell recovery after a short exposure. We selected 10 µM concentration of MSAs to fill most of the binding sites in the assembled MTs [37]. Considering that the MDA binding sites are occluded in the core of the assembled MTs and the effect has to be exerted through the MT ends, we selected lower doses for MDAs (0.1 µM ERIB and MAYT (high toxicity); 0.5 µM for VBL, VCR, and VDS, and 2.5 µM dose of colchicine domain binders). Cells were incubated with MSAs and MDAs for 45 and 35 min, respectively.

Interestingly, our results showed that 13 of the 16 MTAs tested had an effect on the intracellular movement along MT. MDAs were more effective at disrupting the normal function of MTs as cellular roads than MSAs, and apparently, both destabilizing mechanisms (wedging and blocking) were similarly successful. We found some differences within colchicine domain (COL, PPT, MBZ, FBZ, ABZ, and PLIN) or vinca site (VBL, VCR, VDS, and ERIB) binders that could be related to specific physical, chemical, and biochemical properties of each drug. Remarkably, NOS had no effect on KBP movement, which could result from the required metabolic bioactivation of this drug by cytochromes for direct tubulin binding [38].

### 2.3. Biochemical and Structural Characterization of Benzimidazoles Complexes with Mammalian Tubulin

We have seen a clear antiviral effect of benzimidazoles and also activity on MT-mediated transport in mammalian cells. Conversely, these compounds show a strong differential toxicity for helminths, selectively killing them, while exhibiting low or absent mammalian cell toxicity [39]. Therefore, we next focused on understanding the mechanism of action of these drugs and its interaction with mammalian tubulin. We selected MBZ as a representative of the group.

**Figure 5:**
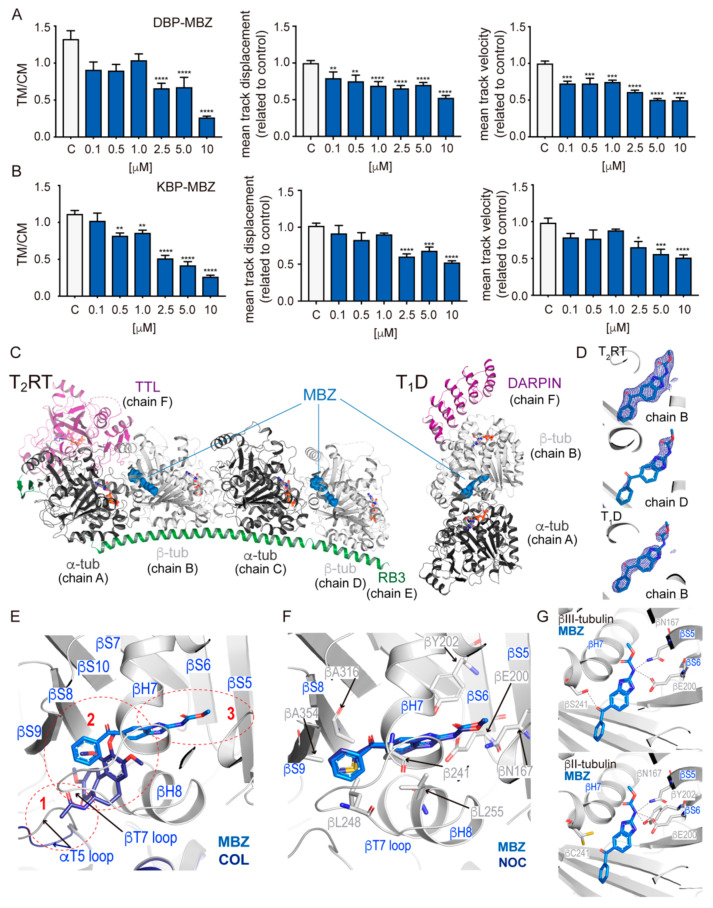
MBZ characterization. (**A**,**B**) DBP and KBP (respectively) particle movement parameters (TM/CM, left; mean track displacement, middle; mean track velocity, right) measured in dose-response experiments using 0.1, 0.5, 1, 2.5, 5, and 10 μM of MBZ in AHRTG cells. Data presented as mean. Error bars indicate S.D. from two independent experiments. Statistically significant differences are indicated by asterisks (**** *p* < 0.0001, *** *p* < 0.001, ** *p* < 0.01, * *p* < 0.05). n = 6. (**C**) Overall structure of T_2_RT (left) and T_1_D (right) tubulin complexes in the presence of MBZ, where proteins are in ribbon representation (α-tubulin in grey, β-tubulin in white, DARPin and TTL in purple, and RB3 in green) and MBZ is represented as spheres bound to the colchicine domain of β-tubulin in blue. (**D**) The sigma A weighted 2mFo-DFc (dark blue) electron density maps are contoured at 1.0 sigma of MBZ (stick representation in blue) at the colchicine domain of each of the protein chains where it was identified. (**E**) Magnified view of the colchicine domain in β-tubulin (ribbon representation in white) showing COL (sticks dark blue) and MBZ (sticks light blue), and highlighting secondary structure elements involved in drug interaction. T5 loop of α-tubulin (ribbon representation) is shown in the open (blue) and close (grey) conformations. The three zones of the pocket are labeled in red. (**F**) Comparison of MBZ (sticks–light blue) and NOC (sticks–dark blue) interaction in the colchicine pocket highlighting secondary structure elements (blue) and main residues (grey) involved in the interaction. (**G**) Hydrogen bonding of MBZ in βIII-tubulin (top) and βII-tubulin (bottom). See crystallographic Table S1.

First, we tested the dose-response of MBZ on tracking experiments using our validated DBP and KBP. We found a clear dose-dependence effect of MBZ on the mean displacement and the mean velocity of DBP, while the ratio TM/CM was only affected at high concentrations (>2.5 μM, Figure 5A). The effect of the drug on the KBP was lower than compared to NOC (a chemically related compound, Figure 5B and Figure S2A), which was unexpected due to the similar binding affinities that we measured for benzimidazoles in the MTC (2-methoxy-5-(2,3,4-trimethoxyphenyl)-2,4,6-cycloheptatrien-1-one) displacement fluorescence assay [40] (Kb 25 °C 5.2 ± 1.4 × 10^5^ M^−1^ for MBZ, 2.1 ± 0.7 × 10^6^ M^−1^ for ABZ and 1.0 ± 0.1 × 10^6^ M^−1^ for NOC). Since NOC shows differences on binding affinity to β-tubulin isotypes [41], we hypothesized that this discrepancy could be related to this differential specificity. Therefore, we next sought detailed structural information of the interaction of MBZ with tubulin using two different macromolecular complexes: T_2_R-TTL, which is made of two αβ-tubulin heterodimers in complex with one molecule of the stathmin-like domain of RB3 and one molecule of the tubulin tyrosine ligase [6]; and the other, T_1_D, which includes one αβ-tubulin heterodimer and a synthetic ankyrin repeat protein, DARPin [42]. We solved the structures of the T_2_R-TTL-MBZ and T_1_D-MBZ complexes and the electron densities refined to 2.20 Å and 2.33 Å resolutions, respectively (Table S1). We found βII tubulin isotype in the T_2_R-TTL complex, and βIII tubulin isotype in the T_1_D complex. The βII- and βIII-tubulin isotypes share an overall sequence identity of 91% [43] with a single difference at the MBZ binding site, where residue 241 (helix H7) is a Cys (βII) or a Ser (βIII).

We unequivocally found MBZ ligand density in chains B of T_1_D and T_2_R-TTL complexes, and at chain D of T_2_R-TTL the density of the ligand was less defined (Figure 5C,D), which correlated with the presence of partial electron density for the βT7 loop. In both structures, MBZ occupies the central pocket of the colchicine domain, as well as a zone buried deeper in β-tubulin and facing the β-sheet of the nucleotide-binding domain [44] (Figure 5E), similarly to the binding mode of NOC (Figure 5F) showing very similar interactions with β-tubulin (Figure 5G), which supports the overall similar binding affinity measured.

MBZ interaction did not affect the overall curved conformation of both tubulin isotypes present either in the T_2_R-TTL or the T_1_D complexes showing an r.m.s.d of 0.22 and 0.35 Å over 370 and 369 Ca-atoms with the related T_2_R-TTL and T_1_D apo-structures (PDB 4i55 and 4drx, respectively). Additionally, these structures displayed a similar conformation to that observed in the presence of other colchicine domain binders bound either to the T_2_R-TTL or the T_1_D complexes. The T_2_R-TTL-MBZ complex have an r.m.s.d. of 0.17 Å (over 375 Ca-atoms) with PDB 4o2b, COL; 0.22Å (over 384 Ca-atoms) with PDB 6y6d, NOS; 0.27 Å (over 376 Ca-atoms) with PDB 5xlt, PPT; 0.24 Å (over 378 Ca-atoms) with PDB 5c8y, PLIN; and 0.18 Å (over 369 Ca-atoms) with PDB 5ca1, NOC. The T_1_D-MBZ complex have an r.m.s.d. of 0.30 Å (over 374 Ca-atoms) with PDB 5nm5, COL, 0.22 Å (over 394 Ca-atoms) with PDB 6s8k, PLIN in βII-tubulin, and 0.20 Å (over 367 Ca-atoms) with PDB 6s8l, PLIN in βIII-tubulin.

The colchicine domain in tubulin can be divided into three zones: a central pocket (zone 2) and two accessory zones, with one facing T5 loop in α-tubulin (zone 1) and the other buried deeper in β-tubulin and facing the β-sheet of the nucleotide-binding domain (zone 3) (Figure 5E) [37]. COL (PDB 4o2b), NOS (PDB 6y6d), and PPT (PDB 5xlt) interact with zones 1 and 2, which involves a change of the α-tubulin T5 loop, which moves to an ‘open’ conformation to make room for these ligands. In our two structures, MBZ occupies zones 2 and 3 and does not contact the α-tubulin T5 loop (which is in a ‘closed’ conformation) and establishes extended interactions with β-tubulin N-terminal (strands S5 and S6) and intermediate (helix H7, H8 and strands S8, S9) domains. Hydrophobic interactions between the benzimidazole moiety and several residues, including L248 (T7 loop), L255 (helix H8), A316 (strand S8), and A354 (strand S9), are closely similar to those found on NOC. However, there are subtle differences specially on the hydrogen bonding interactions. These interactions are established by residues of N167 on S5 and E200 on S6 with the carbamate region (as shown with NOC) and additional residues depending on β-tubulin isotype (Figure 5G).

The mechanism underlying the difference on the effect of benzimidazoles in helminthic vs. mammalian cells remains to be explained. Previous studies showed that the helminthic tubulin-benzimidazole complex is pseudo-irreversible, but not the mammalian one. In fact, although binding is non-covalent, once bound, the ligand cannot be easily removed without denaturing the protein [45]. We then sought the amino acid composition of the colchicine pocket in these invertebrates and compared it to vertebrate’s tubulin binding site (Figure S3) to ascertain the molecular basis of such differences. Remarkably, at position 241, all helminthic tubulins show a Cys (the residue present in βII-tubulin in mammals). Although some residues involved in protein–ligand interaction are highly conserved among helminths and mammals (E200, L248, or L255), there are some differences in the former that could suggest a higher affinity of benzimidazoles for helminthic tubulins. For instance, Y202 is frequently a Phe in helminths and the mutation of this residue back to Tyr (known as F200Y in helminths sequence) has proved to be sufficient for benzimidazole resistance [46]. Notice that this mutation is frequently accompanied by an additional change in the highly conserved residue of E200 (known as E198A in helminths sequence) [47]. Additionally, N167 in mammals can be Ser, Thr, Ala, or even Met in helminths and the mutation into Tyr (known as N167Y in helminths sequence) has been also related to benzimidazoles resistance in *Haemonchus contortus* [48].

## 3. Discussion

### 3.1. MTAs Affect Viral Infections and the Effect Varies According to the Virus Dependency on the Microtubular Network

Our results highlight that targeting the microtubular network with chemical agents directly induces the inhibition of viral replication in five unrelated RNA and DNA viruses that we used as models. However, the inhibitory effect obtained varied depending on the specific functions that viruses have developed throughout evolution to exploit cellular transport machinery. These transport functions might be dependent on the characteristics of particular cell types along with each virus’ susceptibility. Of note is that the dependence on the MT network also varies along the stages of the viral infection cycle. Therefore, variations of the infectivity inhibition are not strictly comparable between viral models and should be considered under each particular circumstance. First, ASFV is characteristically dependent on MT motors (the cellular exit of ASFV virions depends on kinesin-mediated transport [49]) and the ASFV protein p54 hijacks dynein through its light chain DLC1 for intracellular transport [25]. Indeed, peptides designed to bind and compete the interaction site are able to inhibit infection [50]. We have found that MTAs can induce up to 80% of infection inhibition when evaluated with late protein p54 (i.e., accumulation of p54). Nevertheless, the inhibitory effect was lower when infectivity was evaluated with the early expressed protein p30, which denotes that MT function might be dispensable at the early stages of the infection.

It is also known that VACV interaction with MTs is variable as the virus evolves inside cells. At entry, VACV cores move on MTs, but the protein/s responsible are still uncertain (possibly L4R and A10L [51]). Furthermore, immature VACV particles also move from virus factories to sites of wrapping using surface protein A27L, while mature virions move to the plasma membrane to exit using their surface proteins F12L and A36R [52]. The mechanisms elicited involve both molecular motors dynein/dynactin and additionally, specific viral proteins are able to mediate direct binding to tubulin (for association of viral cores with MTs [22]). Given that the replication sites of VACV and ASFV are located at cytoplasmic areas in the vicinity of the nucleus, it is not surprising to find similar results regarding infectivity among both viruses. VACV results were evaluated by detecting infectivity rates using a fluorescent tag expressed under an early/late promoter, which is expected to show the effect of the inhibition at both early and late times of infection.

In contrast with dsDNA viruses, VSV replication can occur anywhere in the cytoplasm. Actin filaments are essential for the incorporation of the nucleocapsids into virions after replication [53]. However, VSV MT-mediated transport is also important [54], but not clearly linked to molecular motors. VSV MT-mediated transport occurs in two phases: shortly after cell entry in particle transport towards the nuclear area and later in a movement directed to the periphery. In fact, our results yielded the highest inhibition by MTAs for VSV compared with other viruses, and MDAs displayed the strongest effect as well.

Finally, the most complex use of cellular filaments might correspond to coronavirus (CoVs), given that actin filaments are principal actors at every stage of the infection. MTs are necessary both for virus internalization and later at several levels of the formation of the viral replication site [55]. In fact, S and M coronavirus proteins interact with tubulin during the infection [56], although their specific function is currently unknown. Such intricate interaction of these viruses with the MT network might explain the weaker, but significant, effect of the MTAs on their infectivity [57]. The multiple interactions between viruses and MTs could implicate that several factors not just limited to movement linked to MT motors might be necessary in order to explain the antiviral effect of MTAs.

### 3.2. MDAs Are More Effective than MSAs in Disrupting Cytosolic Trafficking and Viral Infection

We found that MDAs are overall the best among the MTAs for clinical use on preventing viral infections (Figure 2) and also in disrupting motor protein trafficking (Figure 4). Interestingly, COL has been used during the COVID pandemic to treat the acute respiratory distress syndrome (ARDS), with more than 36 trials registered (in ClinicalTrials.gov accessed on 19 March 2022). This approach was based on the efficiency of COL in several inflammatory diseases. This effect is due to the disruption of the inflammasome activation mediated by mitochondria trafficking on MTs [58].

The imaging tools developed allowed us to monitor cytosolic movement attributed to motor proteins. It is worth noting that some of the movement observed could be partially attributed to endosomal transport, which is also used by several viruses and mediated by motor proteins [59,60,61,62,63,64,65]. These tools have shed light on intracellular transport upon treatment with the clinically available MTAs. From the three parameters analyzed on tracking experiments, the TM/CM ratio was the most affected parameter when using these drugs. MSAs did not affect particle velocity, only PTX significantly decreased the displacement of particles and only CTX affected particle motion (Figure 4C and Figure S2C). Instead, different chemotypes of MDAs proved to significantly affect long-distance cellular transport. Most of the drugs targeting the colchicine domain had a marked effect on reducing TM/CM ratio and particle displacement and velocity apart from NOS. This result was expected, as the AHRTG cell line under study could lack the cytochromes necessary for NOS metabolic bioactivation. In addition, we found that ABZ showed very low effect on motion and no significant modification on particles’ displacement or velocity, opposite to all other benzimidazoles tested. This is likely to be related to ABZ being a pro-drug. Drugs of the vinca domain affected movement very effectively in terms of velocity and displacement and also reduced the TM/CM ratio, with small variations between drugs denoting a similar cellular effect. Finally, MAYT showed a significant reduction of the TM/CM ratio similar to that found for colchicine binders COL or PPT (the most effective ones of this site). Interestingly, the effects on displacement and velocity were even more significant with this drug, denoting that the alteration of tubulin interaction surfaces could be more efficient at disrupting the microtubular movement.

The MDA’s underlying mechanism relies on preventing axial interactions between tubulin heterodimers either by blocking the top interface (maytansine domain) or the essential curve-to-straight conformational change for growing MTs (colchicine and vinca domains). However, we found clear differences among drugs targeting the colchicine or the vinca domains that could be related to variations on the drug–protein interaction networks. These variations would rule the kinetics and thermodynamics of the interaction and control the pharmacological modulation of these drugs. For instance, COL or PPT bind to zones 1 and 2 of the colchicine domain, inducing the flip of β-tubulin T7 loop and α-tubulin T5 loop. In contrast, NOC or MBZ binding to zones 2 and 3 do not involve alterations on the conformation of these loops. Also, their effect varies depending on the cellular area and, for instance, PPT preferably targets MT at the plasma membrane rather than those filaments closer to the nucleus [66]. Moreover, it has been found that NOC does not have a significant effect on MTs built from acetylated α-tubulin of certain cell types [58,67].

Considering that their differential sensitivity underlies the clinical use of benzimidazoles, which is very effective in helminths, and with very few side effects in the mammalian host, we studied their molecular mechanism. Benzimidazoles showed higher affinities for β-tubulin containing a Cys at position 241 (this work [41,43]). Remarkably, helminth’s tubulins include this residue (Figure S3), which together with point modifications in other residues at the binding pocket, could explain a higher affinity of benzimidazoles for helminthic tubulins. Additionally, the differential pharmacokinetic properties of the compounds in helminths and mammals might also be a significant factor in the differential toxicity of these compounds.

In summary, the analysis of additional short-term applications of MTAs on both RNA and DNA viral infections highlighted their activity as broad-spectrum antivirals, including against SARS-CoV-2 infection. Given the variety of processes in which viruses are able to hijack the MT cytoskeleton, the effect of MTAs varied depending on the model virus, the cell type, and the stages of infection analyzed. However, all of them presented antiviral effect to some degree.

To partially understand the cellular basis for the antiviral effect, we studied the movement linked to microtubular motors by time-lapse microscopy using fluorescent-labeled peptides in a cell line with constitutive fluorescent labeling of the microtubular network. These tools allow visualizing the MT function by monitoring the effect of MDAs and MSAs independently. We found that the movement linked to microtubular motors was profoundly affected by MDAs, while MSAs did not modify it to the same extent. Of note is the finding of the strong inhibition induced by colchicine and vinca domains binders, which are compounds with a similar underlying mechanism blocking the curved-to-straight conformational change upon tubulin assembly into MTs. Similarly, the blockage of the top tubulin surface upon polymerization by MAYT seriously decreased MT-based intracellular transport.

Therefore, our assessment of clinically used MTAs support that MTs are a promising target for the development of site-directed antivirals in combinatorial drug therapy. In addition, and considering the wide variety of effects depending on the virus model, new screening tools should be developed to determine which are the most suitable MTAs according to specific viral infections.

## 4. Materials and Methods

### 4.1. Proteins and Chemicals

Calf brain tubulin was purified as described [68] and lyophilized for storage. The stathmin-like domain of RB3, the chicken TTL, and synthetic DarPIN protein preparations were done as described previously [8,69,70]. Paclitaxel (PTX) was from Alfa Aesar Chemical; Docetaxel (DTX) was kindly provided by Rhône Poulenc Rorer, Aventis (Schiltigheim, France); Cabazitaxel (CTX), Ixabepilone (EPO), Nocodazole (NOC), Colchicine (COL), Noscapine (NOS), Podophyllotoxin (PPT), Mebendazole (MBZ), Albendazole (ABZ), Febendazole (FBZ), Vinblastine (VBL), Vincristine (VCR), and Vindesine (VDS) were from Sigma-Aldrich (Saint Louis, MO, USA); Plinabulin (PLIN), Eribulin (ERIB), and Maytansine (MAYT) were from MedChemExpress. The compounds were diluted in 99.8% DMSO-d6 (Merck, Darmstadt, Germany) to a final concentration of 10 mM and stored at −80 °C.

### 4.2. Peptide Synthesis and Labeling

Dynein and kinesin binding peptides (DBP [27] and KBP [28]) were automatically chain assembled by FMOC-SPSS with a peptide synthesizer (Focus XC, AAPPtec, Louisville, KY, USA), using 0.1 mmol of H-Rink-Amide-ChemMatrix resin 0.49 mmol/g, (Biotage, Uppsala, Sweden). Fmoc protected amino acids (Merck) were dissolved in *N*-methylpyrrolidone (Symta, Madrid, Spain) and a standard coupling was performed with *N*,*N*-diisopropylethylamine (Merck) and o-(1-benzotriazol-1-yl)-1,1,3,3-tetramethyluronium hexafluorophosphate, (Merck) for 45 min. For DBP (Ac-CGGHPAEPGSTVTTQNTASQTMSR_8_), R, T, and G were coupled twice, and for KBP (Ac-CGGLEWDDSTLSYR_8_), R, D, and G were coupled twice. Peptides were cleaved with 92.5% trifluoroacetic acid, 2.5% 1,2-ethanedithiol, 2.5% triisopropylsilane, and 2.5% H_2_O for 120 min and then, resins were filtered and trifluoroacetic acid evaporated with a gentle N_2_ flow. The crude peptides were precipitated with cold ether and dissolved in 30% Buffer B (0.1% trifluoroacetic acid, 90% CH_3_CN, 10% H_2_O) in Buffer A (0.05% trifluoroacetic acid in H_2_O) and lyophilized. Peptides were characterized by HPLC-MS equipment from Thermo Fisher (Waltham, MA, USA) coupled to a Finnigan TM LXQ TM detector with electrospray ionization using a Walters column C18 and a gradient of 5 to 95% acetonitrile with 0.1% formic acid.

Labeled Cy5 peptides were prepared as follows: 10 mM Cy5-maleimide in DMSO were added to 6 mg of DBP (dissolved in 50 mM NaHCO_3_ pH 7.0) or KBP (dissolved in 50 mM NaHCO_3_ pH 7.6) to reach a concentration of 1.5 mg/mL of peptide in an equimolar mixture of reagents. The final pH of the reaction was 7.5. The mixtures were incubated under stirring conditions at RT for 1 h. The labeled peptides were purified in a Phenomenex Jupiter Proteo column (Torrance, CA, USA) from a 35–75% gradient of buffer A (2% Acetonitrile, 98% water, 0.1% trifluoroacetic acid) and B (70% acetonitrile, 30% water, 0.09% trifluoroacetic acid). Cy5-DBP and Cy5-KBP eluted at 65 and 63% of buffer B, respectively, and after lyophilization the peptides were obtained with 48 (Cy5-DBP) and 63% (Cy5-KBP) yields. These labeled peptides were dissolved in DMSO to a final 2.5 mM concentration and stored in small aliquots at −20 °C.

### 4.3. Cell Lines and Cultured Conditions

Human non-small cell lung carcinoma A549 cells (ATCC^®^ CCL-185) were used for the development of two new lines, the first one expressing histone H2B-mCherry and α-tubulin-eGFP (cell line AHRTG), while the second expresses angiotensin-converting enzyme (ACE2). Briefly, A549 cells were sequentially transfected to obtain a clone of A549 cells stably expressing both eGFP-α-tubulin and histone H2B-mCherry. Transfection of pH2B_mCherry_IRES_puro2 (addgene #21045) was done using TransIT X2 from Mirus Bio (Madison, WI, USA), and clones were selected in puromycin 0.4 µg/mL (Enzo Life Sciences, Farmingdale, NY, USA) and later checked for fluorescence to select for a further transfection. In a second step, Lipofectamine 3000, from Invitrogen (Waltham, MA, USA), was used for transfection of pIRESneo-EGFP-alpha-Tubulin (addgene #12298), and clones were selected in geneticin G-418 at 1.75 mg/mL (Sigma-Aldrich). The best fluorescent clone was selected, frozen, and freshly thawed vials were used for imaging analysis until we observed a tendency to lose eGFP-α-tubulin.

A549 expressing hACE2 (A549-ACE2) were generated by transduction of parental A549 cells (ATCC CRM-CCL-185) with a retroviral vector expressing human ACE2 [21] and a selection marker that confers resistance to blasticidin. pCMV3-hACE2 was obtained from Sino Biologicals S.L. (Beijing, China).

Vero E6 cells (African green monkey cells, ATCC CRL-1586) and Vero cells (ATCC CCL-81) were obtained from the American Type Culture Collection (ATCC, Manassas, VA, USA), Huh-7 Lunet C3 cells were obtained as a gift from T. Pietschman (Twincore, Germany), and baby hamster kidney cells (BHK-21; 2-14-17 MAW, Kerafast, Boston, MA, USA) were used to obtain rVSV as explained below.

Vero E6, Huh-7 Lunet C3, and A549-ACE2 cells were cultured at 37 °C in Dulbecco’s modified Eagle’s medium (DMEM); (Corning, Corning, NY, USA) supplemented with 100 IU/mL penicillin, 100 µg/mL streptomycin (antibiotics were from Gibco), 10 mM HEPES (Sigma-Aldrich), 1× non-essential amino acids (NEAA, Gibco), and 10% of heat-inactivated fetal bovine serum (FBS) (Gibco). Blasticidin was also included in the medium of A549-ACE2 cells. Vero cells were cultured at 37 °C in DMEM containing 100 IU/mL penicillin, 100 µg/mL streptomycin, 2 mM l-glutamine (Lonza, Basel, Switzerland), and 5% FBS, which was reduced to 2% during viral infection. BHK-21 cells were cultured in DMEM supplemented with 25 µg/mL gentamycin, 2 mM L-glutamine, and 10% FBS. Human non-small cell lung carcinoma A549 cells (ATCC^®^ CCL-185) and AHRTG were cultured in DMEM (Dulbecco’s Modified Eagle Medium; Gibco, Waltham, MA, USA) supplemented with 10% FBS and 1% penicillin-streptomycin. All these mammalian cells were grown at 37 °C and 5% CO_2_ conditions, unless otherwise indicated.

### 4.4. Viruses

SARS-CoV-2 strain NL/2020 (kindly provided by Dr. R. Molenkamp, Erasmus University Medical Center Rotterdam) was propagated in Vero-E6 cells. Infectivity titers were determined in A549-ACE2 cells, using endpoint dilution and immunofluorescence microscopy to determine the number of infectious units (focus forming units, FFU) per ml. Common cold coronavirus 229E expressing GFP (229E-GFP) was kindly given by Volker Thiel, University of Bern, Switzerland. This virus expresses the green fluorescent protein (GFP) instead of ORF4 gene (229E-GFP; [71]) and was used to infect Huh-7 cells (PTA-4583). Recombinant Vesicular Stomatitis Virus (rVSV-Luc) pseudotypes were generated as previously described [72]. BHK-21 cells were transfected to express VSV-G with Lipofectamine 3000 following the manufacturer’s instructions (Invitrogen, Carlsbad, USA). After 24 h, transfected cells were inoculated with a replication-deficient rVSV-Luc pseudotype (multiplicity of infection (moi)1-5 pfu/cell) that contains firefly luciferase instead of the VSV-G open reading frame, rVSV ΔG-luciferase (G* ΔG-luciferase, Kerafast) during 1 h at 37 °C. Next, the inoculum was removed, cells were washed 3 times with phosphate-buffered saline (PBS), and finally, the medium was added. The supernatants were harvested at 24 h, 48 h post-inoculation, centrifuged at 800× *g* for 10 min, and stored at −80 °C. Tissue culture infectious dose per ml was calculated by limiting dilution of each rVSV-Luc virus-containing supernatants on Vero E6 cells (ATCC CRL-1586). Transduction efficiency was measure by luciferase assay (Luciferase Assay System, Promega, Madison, WI, USA).

The recombinant vaccinia virus (VACV) named vtag2GFP containing the tag2GFP under the control of a strong synthetic VACV early/late promoter [73] was kindly provided by Dr. Rafael Blasco (CSIC-INIA, Madrid, Spain).

BPP30GFP is a recombinant African swine fever virus, expressing GFP gene fused to the promoter of the early viral p30 protein [74] Recombinant B54GFP-2 was generated as previously described [75] and expressed GFP as a fusion protein of viral p54, which is an early/late protein that mainly accumulates at the viral replication sites. Both of them were propagated and titrated by plaque assay in Vero cells (ATCC CCL-81) as described [76].

### 4.5. In Cell Motility Assays

Several concentrations of Cy5-DBP and Cy5-KBP, ranging from 1 to 10 µM at several incubation times were tested in AHRTG cells to determine their optimal experimental conditions. AHRTG cells (15,000 per well) were seeded in µ-Slide 8 well ibiTreat (80826, Ibidi, Gräfelfing, Germany) covered with fibrinogen 50 µg/mL (341578, Sigma-Aldrich) at 37 °C in a humid CO_2_ chamber. After 24 h, cells were incubated with 2.5 µM (optimal concentration) of Cy5-DBP or Cy5-KBP for 15 min. The existing medium was replaced by 300 µL of DMEM without phenol red (21063-029, Gibco) and then, cells were imaged using a confocal laser scanning microscope (CLSM) Leica TCS SP8.

AHRTG cells were prepared as explained above and pretreated with different concentrations of MSAs and MDAs compounds or vehicle (DMSO) for 30 min (MSA) or 20 min (MSD). Then, the culture medium was replaced by 250 µL of the drug working solution containing 2.5 µM of Cy5-DBP or Cy5-KBP and incubated for 15 min. Finally, the peptide was washed with drug working solutions, and cells were imaged using a confocal laser scanning microscope (CLSM) Leica TCS SP8 with a 63x oil immersion objective that included a humidified incubation chamber, a CO_2_ controller, and a heating unit. GFP and Cy5 were excited simultaneously at 488 and 646 nm and their emissions were collected at 510–570 and 660–720 nm, respectively. Selected stacks were recorded every 1.793 s for 4 min. Three different fields of each condition were imaged in 2 separate experiments.

Single-peptide trajectories were tracked with the TrackMate plugin from the ImageJ software (https://imagej.net/TrackMate, last accessed on 19 March 2022). Particles with an estimated blob diameter of 1 µm were detected using the LoG detector and linked with the Linear motion LAP tracker. Only spots that were at a maximum distance of 2 µm were linked. Trajectories with less than 5 spots were discarded since they were probably artifactual, and track mean displacement and track mean velocity were calculated and normalized to control cells. Data were further analyzed with MATLAB to perform a Mean Square Displacement (MSD) analysis to determine the mode of displacement of the peptides followed over time [77,78] Briefly, for each trajectory the MSD function was calculated and its log-log representation fitted with a linear function ($log\left(MSD\left(\tau \right)\right)\;=\;2\times log\left(\tau \right)\;+\;C)$. MSD curves with an R^2^ coefficient <0.8 were removed. According to the modeling study by Qian et al. [79], the slope alpha of each MSD curve determines the motion type of each particle, so particles were classified according to the alpha value of each trajectory: constrained particles (CM) α < 1, Brownian particles (BM) α = 1, and transported particles (TM) α > 1. Two independent experiments were performed using 3 biological replicates (N = 6). Data were analyzed by one-way ANOVA using GraphPad Prism 8 software (San Diego, CA, USA). For multiple comparisons, Bonferroni’s correction was applied. Values were represented in a bar graph as mean. Error bars indicate S.E.M. from two independent experiments. Statistically significant differences are indicated by asterisks (**** *p* < 0.0001, *** *p* < 0.001, ** *p* < 0.01, * *p* < 0.05).

### 4.6. Drug Treatment and Cell Viability Determination

We tested the effect of the following MSA- or MDA-approved compounds in coronavirus infection. To determine experimental working concentrations, cell viability was first analyzed for each drug after 24 h of treatment in cytotoxicity assays using CellTiter 96 Non-radioactive Cell Proliferation Assay (Promega) and following the manufacturer’s instructions. Based on these results, we selected the highest non-toxic concentrations (nM) for the experiments on Huh-7 Lunet C3, Vero E6, and Vero cell lines, shown in Table 1.

A549-ACE2 cells were subjected to MTT assays following standard procedures after 48 h of treatment [80]. Concentrations depicted in Figure 5 are shown on Table 1. All concentrations tested were 0.5× CC50, 1× CC50, 2× CC50 (Figure S1): PTX (25, 50, and 100 nM), DTX (12.5, 25, and 50 nM), CTX (0.5, 1 and 2 µM), EPO (12.5, 25, and 50 nM), COL (25, 50, and 100 nM), NOS (12.5, 25, and 50 µM), PPT (25, 50, and 100 nM), MBZ (0.5, 1, and 2 µM), FBZ (0.5, 1, and 2 µM), ABZ (0.5, 1, and 2 µM), PLIN (0.5, 1, and 2 nM), VBL (25, 50, and 100 µM), VDS (1.5, 3, and 6 nM), VCR (100, 200, and 400 nM), ERIB (2.5, 5, and 10 nM), and MAYT (5, 10, and 20 nM).

### 4.7. Infection Assays

Huh-7 Lunet C3 or Vero cells were pretreated with compounds for 1 h at 33 or 37 °C, followed by infection with 229E-GFP or ASFV recombinants, respectively, at a moi of 1 pfu/cell. Cells were washed twice with growth medium after 90 min of adsorption at 33 or 37 °C and then incubated with fresh medium containing the compounds for 24 h. Cells were then harvested with PBS-EDTA 5 mM or Trypsin-EDTA (Gibco) respectively, and diluted in PBS. Detection of infected cells was performed by analyzing GFP expression. To determine the percentage of infected cells per condition, 10,000 cells per time point were scored and analyzed in a FACS Canto II flow cytometer (BD Sciences, Franklin Lakes, NJ, USA). Infected cell percentages obtained after drug treatments were normalized to DMSO values.

For r-VSV-Luc pseudotype experiments, Vero-E6 cells were seeded onto 96-well plates the day before. Compounds were diluted in complete media to achieve the final concentration. Compound dilutions were applied to the cell cultures 1 h before the virus inoculation. Pretreatment was removed and fresh compound dilutions containing the rVSV-Luc pseudotype were used to inoculate the cultures for 24 h. A total of 24 h post inoculation, cells were lysed for luciferase activity determination using Steady-Glo Luciferase Assay System (Promega) and luminescence was quantified in the EnSight multimode plate reader of PerkinElmer (Waltham, MA, USA). Relative infection values were determined by normalizing the data to the average relative light units detected in DMSO-treated cells. SARS-CoV-2 strain NL/2020 infection was estimated by immunofluorescence microscopy and automated imaging. A549-ACE2 cells were seeded onto 96-well plates using 2 × 10^4^ cells/well. The following day, cultures were inoculated with SARS-CoV-2 (moi 0.01) in the presence of the compound doses corresponding to 2×, 1×, and 0.5× as the measured CC50 in A549-ACE2 cells. A total of 48 h post infection, cells were fixed for 30 min at RT with a 4% formaldehyde solution in PBS, washed twice with PBS, and incubated with incubation buffer (3% BSA; 0.3% Triton X100 in PBS) for 1 h. A monoclonal antibody against S protein was diluted in incubation buffer (1:2000; a generous gift from Luis Angel Fernández and Jose María Casasnovas-CNB) and incubated with the cells for 1 h, after which time, the cells were washed with PBS and subsequently incubated with a 1:500 dilution of a goat anti-human conjugated to Alexa 488 (Invitrogen-Carlsbad). Nuclei were stained with DAPI (Life Technologies, Carlsbad, CA, USA) during the secondary antibody incubation following the manufacturer’s recommendations. Cells were washed with PBS and imaged using an automated multimode reader (TECAN Spark Cyto, Männedorf, Switzerland). The percentage of SARS-CoV-2-positive cells was determined in each well by automated segmentation and positive-versus-negative discrimination using cells infected in the presence of the vehicle and mock-infected cells as controls. Two independent experiments were performed using 3 biological replicates (N = 6). Data were analyzed by one-way ANOVA using GraphPad Prism 8 software (San Diego). For multiple comparisons, Bonferroni’s correction was applied. Values were represented by bars graph as mean ± SD of at least three independent experiments, unless otherwise stated. A *p*-value ˂ 0.05 was considered as statistically significant.

### 4.8. Crystallization and Crystal Structure Determination

Tubulin was resuspended in 15 mM MES, pH 6.8, 0.5 mM EGTA, and 0.1 mM GTP, equilibrated through a G-25 column and centrifugated at 50 K r.p.m. for 10 min at 4 °C to remove aggregates. The T_1_D complex was formed by mixing tubulin and DARPin in a 1:1.5 ratio in a buffer containing 0.5 mM MgCl_2_ and a final GTP concentration that was 5 times the tubulin concentration. The mixture was incubated 15 min on ice and subsequently gel filtrated in a Superdex 200 column in buffer 15 mM Pipes, pH 6.8, 0.2 mM EGTA, 0.3 mM MgCl_2_, and 0.2 mM GTP. T_1_D complex was concentrated (Amicon MWCO 10) to 20 mg/mL and flash frozen in liquid nitrogen. Native T_1_D complex was crystallized by hanging drop vapor diffusion at 20 °C in a reservoir volume of 500 μL of 0.1 M Bis-Tris Methane, pH 5.5, 0.2 M Ammonium Sulphate, 18–22% PEG 3350, mixing 1 μL of complex and 1 μL of reservoir solution. Suitable crystals were exchanged into reservoir solutions containing 1–2 mM MBZ and soaked for 15 to 30 min. Before flash-cooling in liquid nitrogen, crystals were cryo-protected using 20% glycerol. The T_2_R-TTL complex was prepared as described [81] and supplemented with 10 mM DTT, 0.1 mM GDP, and 1 mM AMPCPP before setting crystallization experiments. The native T_2_R-TTL complex was crystallized by hanging drop vapor diffusion at 20 °C with a reservoir volume of 500 μL of 0.1 M MES/0.1 M Imidazole pH 6.5, 0.03 M CaCl_2_/MgCl_2_, 5 mM L-Tyr, 8% glycerol, 5.5% PEG 4000, mixing 1 μL of complex, and 1 μL of reservoir solution. Suitable crystals were exchanged into reservoir solutions containing 1–2 mM MBZ and soaked for 15 to 30 min. Prior to flash-cooling in liquid nitrogen, crystals were cryo-protected using 10% PEG 4000 and increasing glycerol concentrations (16 and 20%).

X-ray diffraction data were collected at beamline XALOC at ALBA Synchrotron (Spain). Diffraction intensities were indexed and integrated using XDS [82] and scaled using AIMLESS [83]. Molecular replacement was performed with PHASER [84] using the previously determined structure (PDB 5nm5 for the T_1_D complex and PDB 4o2b for the T_2_R-TTL complex) as a search model. Interestingly, the density of the side chains found indicates that the complex preparation method preferably selects the βII tubulin isotype in the case of the T_2_R-TTL complex and the βIII isotype in the T_1_D complex. Structures were completed with cycles of manual building in COOT [85] and refinement in PHENIX [86]. Figures were prepared in Pymol (Schrodinger). Data collection and refinement statistics are given in Table 1. The atomic coordinates were deposited in the Protein Data Bank (https://www.rcsb.org/ accessed on 2 May 2021) with PDB codes 7odn and 7ogn.

## Figures and Tables

**Table 1:** Concentrations of clinically approved used in the infection assays.

	Huh-7 Lunet C3	Vero E6	Vero	A549-ACE2
paclitaxel (PTX)	5	25	25	0.025
docetaxel (DTX)	0.03	10	10	0.0125
cabazitaxel (CTX)	0.01	25	10	0.5
ixabepilone (EPO)	0.05	25	25	0.0125
colchicine (COL)	0.2	25	75	0.1
noscapine (NOS)	100	100	100	50
phodophyllotoxin (PPT)	0.2	50	50	0.1
mebendazole (MBZ)	5	10	10	2
albendazole (ABZ)	1	25	25	2
febendazole (FBZ)	1	50	75	2
plinabulin (PLIN)	0.5	25	25	0.002
vinblastine (VBL)	0.05	5	25	100
vincristine (VCR)	5	1	1	0.4
vindesine (VDS)	0.02	100	100	0.006
eribulin (ERIB)	0.01	10	100	0.0025
maytansine (MAYT)	0.1	25	25	0.02

## Data Availability

The atomic coordinates of βII tubulin isotype-MBZ complex (T_2_R-TTL complex) and βIII tubulin isotype-MBZ complex (T_1_D complex) have been deposited in the Protein Data Bank (https://www.rcsb.org/ accessed on 2 May 2021) with PDB codes 7odn and 7ogn.

## Supplementary Materials

The following supporting information can be downloaded at: https://www.mdpi.com/1422-0067/23/7/3448/s1.
