# Supplementary material for: Effect of Clinically Used Microtubule Targeting Drugs on Viral Infection and Transport Function
Source: Int J Mol Sci. 2022 Mar 22;23(7):3448. doi: 10.3390/ijms23073448 (PMC8998746; doi:10.3390/ijms23073448)
Supplement: Supplementary file 1 [file ijms-23-03448-s001.zip › 2021 Oliva et al e SI IMJS.pdf]

Table S1: Crystallographic table (Figure 4)

	Native TD1-MBZ PDB 7odn*	Native T2RT-MBZ PDB 7ogn*
**Data collection**		
Space group	$P2_1$	$P2_12_12_1$
Cell dimensions		
a, b, c (Å)	73.441, 91.390, 82.671	104.900, 158.010, 179.689
α, β, γ (°)	90.00, 97.234, 90.00	90.00, 90.00, 90.00
Resolution (Å)	82.13 – 2.33 (2.41 – 2.33) **	50.35 – 2.20 (2.24 – 2.20)**
Total reflections	189889 (18547)	1440160 (72994)
Unique reflections	45660 (4418)	150908 (7375)
Redundancy	4.2 (4.2)	9.5 (9.9)
Completeness (%)	98.5 (97.9)	99.6 (98.8)
Mn (I/sd)	11.9 (1.8)	16.7 (1.7)
CC_{half}	0.997 (0.865)	0.999 (0.670)
R_{merge}	0.081 (0.796)	0.074 (1.354)
R_{pin}	0.045 (0.441)	0.025 (0.446)
R_{meas}	0.093 (0.913)	0.078 (1.427)
Wilson B factor	36.3	45.6
**Refinement**		
Resolution (Å)	49.109 – 2.33	50.349 – 2.20
No. of reflections	45571	150804
R_{work}/R_{free}	0.1882/0.2211	0.2087/0.2341
No. non-hydrogen atoms	8136	34986
Protein	7905	34337
Ligand	122	422
Water	109	227
Average B-factor	51.1	65.1
Protein	51.2	65.5
Ligand	51.5	56.2
Water	41.9	46.3
r.m.s deviation		
Bond lengths (Å)	0.0018	0.002
Bond angles (°)	0.56	0.53
Ramachandran statistics (%) (allowed/favored/outliers)	97.99/2.01/0.00	98.03/1.97/0.00
Rotamer outliers (%)	0.00	0.00
Clashscore /MolProbity Overall score	1.14 / 0.83	0.66 / 0.72

* Data were collected from one crystal ** Values in parenthesis are for highest-resolution shell

Supplemental Figure S1

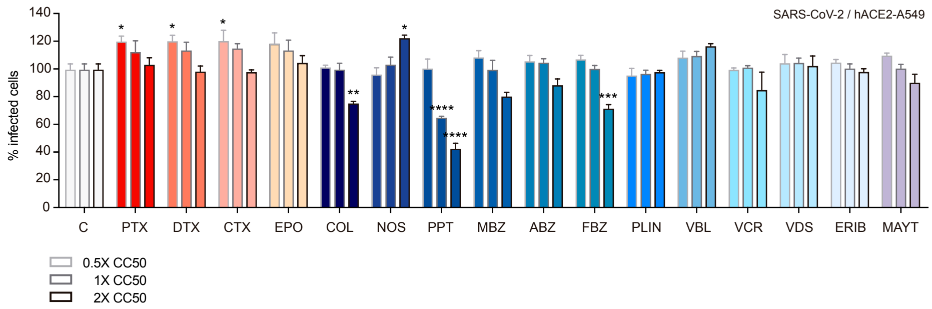

Figure S1: SARS-CoV-2 additional comparison of several doses. SARS-CoV-2 infection efficiency in the presence of the compound doses corresponding to 0.5X, 1X and 2X CC50 (gray to black rim columns). A549-ACE2 cells were inoculated with SARS-CoV-2 (moi 0.01) after one hour of pretreatment with different compound doses. Infection efficiency estimation was measured by immunofluorescence microscopy and automated imaging 48 h post infection. The percentages of efficiency have been normalized to DMSO values. Data presented as mean. Error bars indicate S.D. from two independent experiments (one-way ANOVA with Bonferroni post-test). Statistically significant differences are indicated by asterisks (****$p < 0.0001$, ***$p < 0.001$, ** $p < 0.01$, *$p < 0.05$), n=6.

Supplemental Figure S2

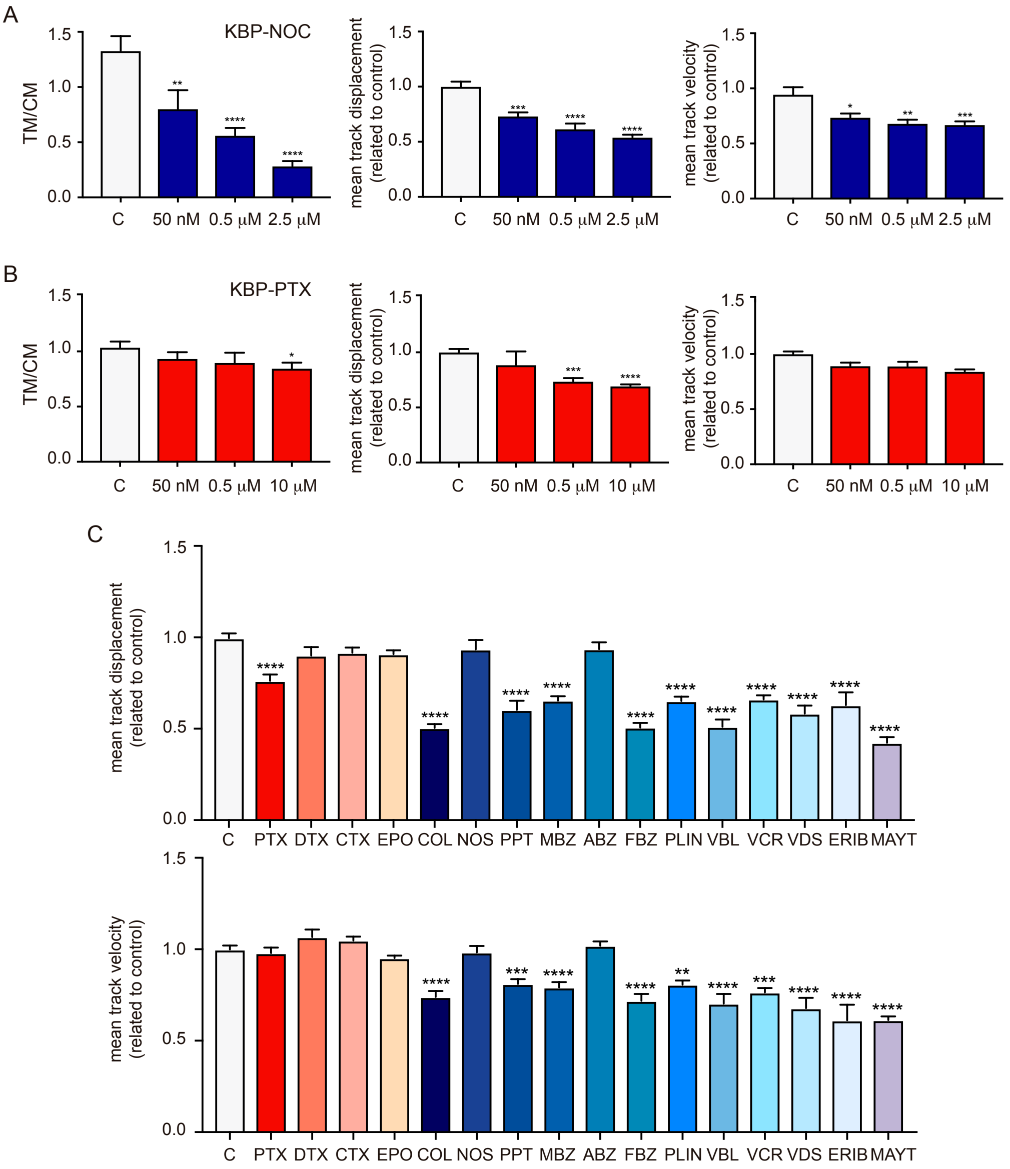

Figure S2: KBP particle movement parameters measured in dose response experiments with NOC **(A)** and PTX **(B)** in AHRTG cells. TM/CM (left), mean track displacement (center) and mean track velocity (right), analyzed using the TrackMate plugin in ImageJ. **C.** KBP mean track displacement (top) and mean track velocity (bottom) parameters measured upon pharmacological treatment with clinically used MTAs. Data presented as mean. Error bars indicate S.D. from two independent experiments (one-way ANOVA with Bonferroni post-test). Statistically significant differences are indicated by asterisks (****$p < 0.0001$, ***$p < 0.001$, **$p < 0.01$, *$p < 0.05$), n=6.

Supplemental Figure S3

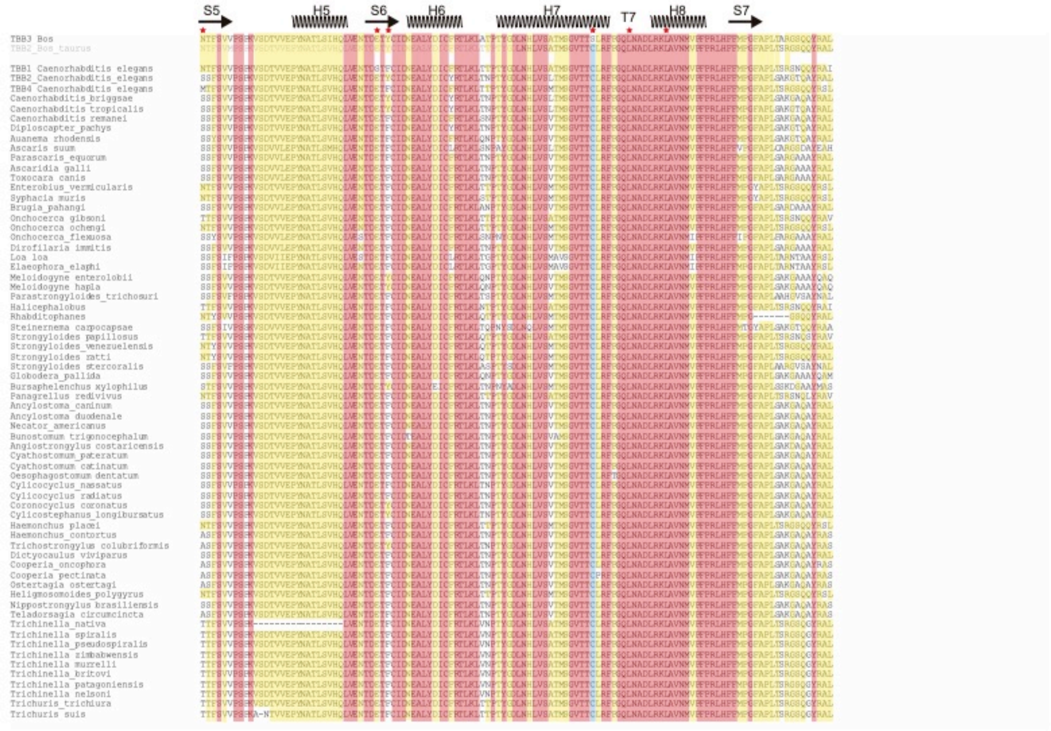

Figure. S3: Helminth tubulins alignment compared to mammalian βIII- and βII-tubulins (represented by *Bos taurus* used in the macromolecular crystallography experiments). Secondary structural elements of tubulin are highlighted above the sequences and, red stars show main residues involved in the interaction with MBZ. Highly conserved residues among all sequences (helminths and mammals) are shown in red, in yellow those that are shared by most of the sequences but are not fully conserved, and C241 is depicted in blue. The colchicine binding pocket shares the C241 at the binding site of studied benzimidazoles as found in mammals βII-tubulin isotype, whereas in that position there is a Ser in isotype βIII-tubulin. There are further variations on several residues involved in benzimidazoles interactions, such as N167 at S5 or E200 and Y202 at S6.
